# Responsible gambling through a motivational telephone intervention to high-risk gamblers – An evaluation of user satisfaction and subjective intervention effects

**DOI:** 10.3389/fpsyg.2022.917216

**Published:** 2022-11-30

**Authors:** Anders Håkansson, Katja Franklin, Maria Dahlström, Axel Lyckberg

**Affiliations:** ^1^Department of Clinical Sciences Lund, Psychiatry, Faculty of Medicine, Lund University, Lund, Sweden; ^2^Region Skåne, Gambling Disorder Unit, Malmö, Sweden; ^3^Region Skåne, Competence Center Addiction, Malmö, Sweden; ^4^AB Svenska Spel, Visby, Sweden

**Keywords:** gambling disorder, motivational interviewing, responsible gambling, problem gambling, online gambling

## Abstract

**Background and aims:**

Problem gambling causes major social and psychiatric consequences, and there is need to develop feasible harm-reducing or therapeutic interventions. It has been suggested that gambling operators with responsible gambling intentions can intervene in order to identify and help reduce the harm in problem gamblers. However, user satisfaction, and the risk of negative reactions and gamblers’ transfer to other operators, rarely have been explored scientifically.

**Methods:**

This is a retrospective survey study of gamblers reached by a motivational, telephone intervention by the Swedish state-owned gambling operator (Svenska Spel), addressing gamblers with indicators of high-risk gambling practices (*n* = 197). Surveys were answered approximately 10 days after the intervention.

**Results:**

Thirty-seven percent perceived their gambling to have decreased due to the intervention, whereas 7% perceived their gambling instead had increased. A large majority (80%) reported a subjective favorable experience from the intervention. Forty percent reported gambling on other operators than Svenska Spel after the intervention, but only 7% gambled on a new operator following the intervention.

**Conclusion:**

Survey data in gamblers reached with a motivational telephone intervention present mainly promising findings, and should be replicated in future studies in larger samples. The main findings display an overwhelmingly favorable subjective experience, and a limited risk of gamblers migrating to other operators.

## Introduction

Problem gambling presents a health hazard world-wide, with severe social and mental health-related consequences, but is also a condition which can be prevented or treated (Potenza et al., 2019; Di Nicola et al., 2020). A number of low-threshold harm-reducing or therapeutic interventions have been suggested (Gainsbury et al., 2020), including those delivered directly by gambling operators with responsible gambling policies (Jonsson et al., 2019). These may, for example, involve mandatory play breaks, direct automated messaging with personalized feedback on gambling patterns (Hopfgartner et al., 2021), or general consumer protection information providing links to different harm-reducing measures, such as voluntary self-exclusion or limit setting (Gainsbury et al., 2020). However, a more direct contact from a gambling operator, such as in a personalized telephone call aiming to reach gamblers at risk with responsible gambling measures, is far less common (Jonsson et al., 2019).

As part of its responsible gambling strategy, the state-owned gambling operator AB Svenska Spel, in Sweden, has introduced systematically a motivational telephone intervention to gamblers who show signs of high-risk or harmful gambling. The intervention is aimed for clients of Svenska Spel’s Sports & Casino sub-section, which acts as an independent operator in the market of sports betting, horse race betting, online casino, online bingo, and online poker. The gamblers included in this study, who received a telephone call, were either gamblers who completed a voluntary self-assessment test which concluded that their level of gambling was problematic (GamTest, Jonsson et al., 2017, 2019; Forsström et al., 2020), or gamblers who had accumulated substantial losses; above 100,000 SEK (around 9,000 Euros) over 12 months, and above 10,000 SEK (around 900 Euros) in 1 month for gamblers aged 25 or younger. The present intervention project has been described previously (Håkansson et al., 2020).

In a similar project in Norway, the state-owned gambling operator carried out motivational contact attempts addressing subjects with intense gambling practices, in that case defined as the proportion of the operator’s clients with the highest net losses. Here, a personal telephone intervention was more efficacious in reducing high-risk gambling practices, compared to a postal letter contact or a control group (Jonsson et al., 2020, 2021). These prior analyses, however, are limited by the fact that only the own operator’s gambling data can be measured. Thus, even in subjects with reduced or discontinued gambling on the gambling operator which intervenes, it is still possible that further gambling may occur at other operators. Likewise, studies hitherto have not been able to assess, retrospectively, how included individuals perceive and experience the intervention, and which subjective effects the intervention may have on gambling behaviors.

For example, the gambling market in Sweden involves a larger number of licensed gambling operators, many of which offer online games involving either online casino or online sports betting, i.e., gambling types which are easily accessible to individuals who may increase their gambling practices on other operators, after receiving a responsible gambling intervention in one operator. It shall be borne in mind, for example, that when gamblers are self-excluded or otherwise prevented from gambling on one operator, people with high-risk gambling practices instead may turn to other online operators thereafter. This was seen in a recent study on a specific regulation during COVID-19, where a deposit limit, and a time limit, were applied to online casino gambling and electronic gambling machines. Among gamblers excluded after reaching these limits, many of them continued to gamble on other operators (Håkansson et al., 2022). Also, although it may not be common (Ivanova et al., 2019), it is also possible that gamblers reached with a motivational intervention may react with anger, potentially with a deteriorating effect on their gambling practices. In particular, while responsible gambling tools have not been shown to cause irritation and to scare off gamblers in the segment of non-problem and moderate-risk gamblers, such negative reactions were shown to be significantly more common in problem gamblers (Ivanova et al., 2019). Therefore, in an intervention aiming to help high-risk gamblers reduce or discontinue their gambling, it is of utter importance to assess individuals’ subjective experience of such interventions.

For these reasons, the present study was carried out in gamblers who had been reached by AB Svenska Spel in a motivational telephone intervention. This survey study aimed to assess the following measures in gamblers reached by this intervention; (1) subjective user satisfaction with the intervention, (2) subjective opinion about how the intervention had changed their gambling behavior following the intervention, (3) gambling on the own operator and on other gambling operators following the intervention, including the potential risk of migrating to a new gambling operator after the present responsible gambling intervention, and (4) whether user satisfaction with the intervention was associated with changes in gambling and potential gambling on other operators after the intervention.

## Materials and methods

The study involved individuals who had undergone the motivational intervention (Håkansson et al., 2020), either based on a “red” self-test (GamTest, Forsström et al., 2020), or based on high levels of detected gambling losses (although cut-off levels have varied during the study period, as previously reported Håkansson et al., 2020). In brief, the calls, following an overall motivational interviewing model (Arkowitz and Miller, 2008), contained the following components:

- Information about the intervention and permission to proceed
- Questions and information about the client’s losses and her/his reactions to this information
- Rolling with resistance, a typical component in motivational talks (Arkowitz and Miller, 2008), when the client expresses any opposition to describing the gambling habits as problematic
- Reflective listening and continuation of the call depending on the client’s motivation and depending on the client’s “change talk,” which is a key component in a motivational conversation (Gaume et al., 2021)
- In case of the client’s permission, further information and discussion about responsible gambling tools such as limit settings or self-exclusion
- A discussion around the client’s plans for future gambling behaviors.

**Figure 1:**
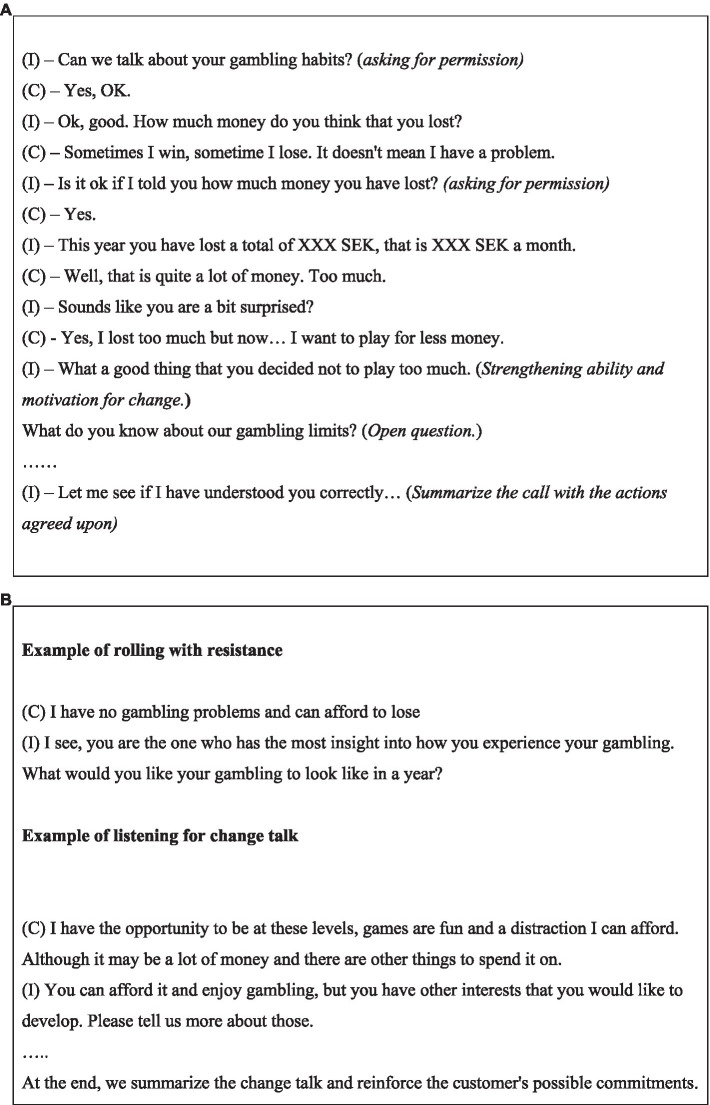
**(A)** Example of motivational intervention call, in a shortened and simplified dialogue format between interviewer (I) and client (C). **(B)** Examples of “rolling with resistance” and “listening to change talk” as within the motivational intervention model.

**Figure 2:**
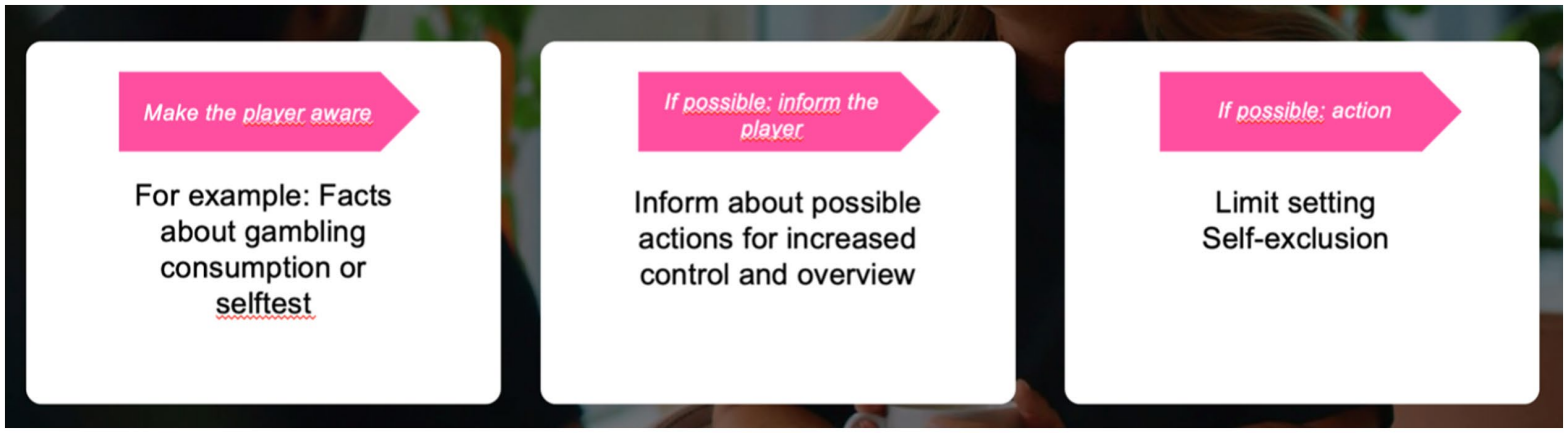
Stylistic structure for the planning of motivational intervention calls.

The telephone conversations had a motivational, non-judgmental, and reflective listening approach. For an example of how a motivational call could be conducted, see Figures 1A,B, 2. Study subjects were contacted 10 days after the call. The study was carried out from March 23, 2021, through October 1, 2021. A total of 900 subjects were invited, and 204 complete responses (and 39 partial responses) were collected.

Surveys were opened only in case an individual provided informed consent in the electronic format of the survey. For a completed study response, an individuals received a compensation consisting of an electronic gift card with a value of 100 SEK, corresponding to around nine Euros (a gift card possible to use in a number of stores, although not for the purchase of alcohol, tobacco, or gambling).

In several cases, an individual had responded both with a complete or a partial survey response, i.e., more than once. Here, only the first survey was kept, and thereafter, the final sample size was 197 individuals with one complete study answer.

The study was approved by the Swedish Ethics Authority (application file number 2020-03281). It was presented in a protocol paper (Håkansson et al., 2020), and pre-registered at clinicaltrials.gov (identifier NCT04646421).

### Measures

Questions referred to the telephone intervention carried out, and the items describing the subject’s subjective experience of the intervention were based on the following questions, which were rated on a 5-point scale ranging from “fully agree” to “fully disagree” (Table 1): the telephone call (1) makes me feel the operator cares about me, (2) makes me angry or annoyed, (3) made me decrease my gambling after the call, (4) made me increase my gambling after the call, (5) gave me primarily a favorable experience, and (6) gave me primarily an unfavorable experience.

**Table 1:** Subjective patient satisfaction with the motivational telephone intervention (*N* = 197).

	Call makes me feel the operator cares about me, *n* (%)	Call makes me annoyed or angry, *n* (%)	Call decreased my gambling, *n* (%)	Call increased my gambling, *n* (%)	Call gave me primarily a favorable experience, *n* (%)	Call gave me primarily an unfavorable experience, *n* (%)
Fully agree	132 (67)	8 (4)	20 (10)	7 (4)	103 (52)	6 (3)
Partly agree	42 (21)	18 (9)	53 (27)	6 (3)	54 (27)	9 (5)
Neither	13 (7)	26 (13)	69 (35)	45 (23)	29 (15)	33 (17)
Partly disagree	5 (3)	26 (13)	15 (8)	24 (12)	8 (4)	34 (17)
Fully disagree	5 (3)	119 (60)	40 (20)	115 (58)	3 (2)	115 (58)

The subsequent questions asked whether the subject, since the telephone intervention, had self-excluded on the nationwide Swedish self-exclusion service “Spelpaus” (Håkansson and Henzel, 2020). The self-exclusion service is national, managed by a governmental state-wide authority, and it enables any individual in Sweden to self-exclude from all licensed gambling operators in Sweden for one or several periods of 1, 3, 6 or 12 months. This voluntary self-exclusion system technically excludes enrolled individuals from both land-based and online gambling operators, except for lottery tickets bought without personal registration in grocery stores and similar, and the relatively minor market of land-based bingo venues. The present self-exclusion system is likely one of very few multi-operator self-exclusion services operating in a whole country, for a large number of gambling types, and independently from each individual operator (Håkansson and Henzel, 2020).

Other questions addressed whether the respondent had gambled on a different gambling operator than Svenska Spel, after the telephone intervention, and if so, whether the subject had gambled on an operator on which she/he had never gambled before. Thereafter, the subject was asked about which gambling types she/he had gambled at any time during the past year prior to the intervention, and at any time after the telephone intervention, respectively. Gambling types were collapsed into the categories of sports betting (on any operator), horse race betting (on any operator), online poker gambling (on any operator), and online casino/bingo gambling (including online bingo and online casino on any operator, Table 2). The following items asked about whether the subject had gambling-related debts, whether she/he had any involvement with the Swedish enforcement agency due to debts, and questions about current occupation (work, studying, retired, sick-leave, or unemployed), gender (female/male), and age group (<25, 25–29, 40–59, or >59 years).

### Statistical methods

A comparison was made between subjects who reported having self-excluded from gambling after the intervention versus those who had not, between those reporting decreased gambling after the intervention vs. all others, those reporting gambling on other operators after the intervention vs. others, and between those reporting gambling on a new operator after the intervention vs. all others. These comparisons were made using Chi-square tests (Fisher’s exact whenever the number in any of the groups was five or lower). Regarding self-reported decrease in gambling after the intervention, and regarding any gambling on other operators after the intervention, these outcome variables were analyzed in logistic regression analyses, including age group, gender, and each of the gambling types for which the individuals could have received the motivational intervention, i.e., the gambling types provided by the operator Svenska Spel Sport & Casino (online casino/bingo gambling, sports betting, horse race betting, and online poker). Apart from these gambling types, other gambling types are reported for descriptive purposes in Table 2. Due to the limited statistical power and the low number of individuals endorsing some outcomes, no logistic regression analysis was carried out for gambling on a new operator after the intervention, for the reporting of having self-excluded after the intervention, or for the perception of having gambled more after the intervention.

**Table 2:** Self-reported gambling patterns for the past year prior to the intervention, and for the time period between the intervention and the survey (approximately 10 days), respectively (*N* = 197).

	Past-year gambling prior to the intervention, *n* (%)	Gambling after the intervention, *n* (%)
Sports betting on Svenska Spel	176 (89)	156 (79)
Sports betting on other operators	102 (52)	71 (36)
Horse race betting	97 (49)	63 (32)
Svenska Spel online casino	93 (47)	70 (36)
Other operators’ online casino	81 (41)	52 (26)
Land-based casino	19 (10)	4 (2)
Online poker	45 (23)	30 (15)
Online bingo	25 (13)	9 (5)
Land-based bingo	4 (2)	5 (3)
Restaurant casino	16 (8)	8 (4)
Land-based electronic gambling machines	44 (22)	21 (11)

## Results

A majority of included individuals were male (93%, *n* = 183), and working (75%, *n* = 147) or studying (10%, *n* = 20), whereas 7% (*n* = 13) were retired, 5% (*n* = 10) were unemployed, and 4% (*n* = 7) were on sick-leave. A total of 16% (*n* = 32) had gambling-related debts, and 17% (*n* = 34) had debts involving the national enforcement authority. The largest age groups were 40–59 years (42%, *n* = 83), and 25–39 years (26%, *n* = 51), respectively, whereas 19% (*n* = 38) were younger than 25 years, and 13% (*n* = 25) were 60 years or older.

A large majority of respondents (88%) perceived the telephone intervention as a sign of the operator caring about the gambler, and a minority, 13% of respondents, endorsed being annoyed or angry with the intervention. Regarding gambling after the intervention, 37% reported that they perceived their gambling to have decreased because of the intervention, whereas a minority (7%) reported that their gambling had increased because of the intervention. A large majority (80%) reported a mainly favorable experience of the telephone intervention, and few (8%) a mainly unfavorable experience (Table 1).

Gambling data reported for the past-year period prior to the intervention, and for the period after the intervention, respectively, are reported in Table 2.

Seven percent (*n* = 13) reported that they had self-excluded from gambling after the intervention. Those who had self-excluded were significantly younger (*p* = 0.03, Chi-square linear-by-linear, with 31% in the youngest age group versus 18% among the remaining respondents), whereas they did not differ significantly with respect to the number of sports bettors (85 vs. 91%, *p* = 0.36, Fisher’s exact test), online casino/bingo gamblers (69 vs. 52%, *p* = 0.26 Fisher’s exact test), horse race bettors (54 vs. 49%, *p* = 0.78, Fisher’s exact test), online poker gamblers (23 vs. 23%, *p* = 1.00, Fisher’s exact test), or men (85 vs. 93%, *p* = 1.00, Fisher’s exact test).

Forty percent (*n* = 79) reported that they had gambled on a different gambling operator (than Svenska Spel) after the intervention. Among them, 16% (*n* = 13, 7% of all respondents) reported that they had gambled on an operator on which they had never gambled before. Those who had gambled on a new operator after the intervention were significantly younger (*p* < 0.001, Chi-square linear-by-linear, with 54% in the youngest age group versus 17% among the remaining respondents), whereas they did not differ significantly with respect to the number of sports bettors (77 vs. 91%, *p* = 0.12, Fisher’s exact test), online casino/bingo gamblers (77 vs. 52%, *p* = 0.09 Fisher’s exact test), horse race bettors (31 vs. 51%, *p* = 0.25, Fisher’s exact test), online poker gamblers (15 vs. 23%, *p* = 0.74), or men (92 vs. 93%, *p* = 1.00, Fisher’s exact test).

Gamblers who reported having mainly a favorable experience of the telephone intervention were significantly more likely to report that their gambling decreased after the intervention (43 vs. 15%, *p* < 0.01), and less likely to report gambling on other operators after the intervention (36 vs. 55%, *p* = 0.03). They did not differ significantly from other subjects with respect to self-exclusion after the intervention (8 vs. 3%, *p* = 0.24) or the reporting of having gambled on a new gambling operator after the intervention (5 vs. 13%, *p* = 0.09). Gamblers who reported being angry or annoyed by the call tended to be more likely to report gambling on an operator on which they had never gambled before (15 vs. 5%, *p* = 0.05), but were not more likely to report decreased gambling (39 vs. 37%, *p* = 0.87), self-exclusion (8 vs. 6%, *p* = 0.81), or any gambling on a different operator than Svenska Spel (39 vs. 40%, *p* = 0.85).

In logistic regression, a self-reported decrease in gambling after the intervention was not significantly associated with the gender (*OR* 0.49 for male gender [0.12–2.06], *p* = 0.33), age group (*OR* 0.98 [0.69–1.38], *p* = 0.89), or with any of the specific gambling types assessed (*OR* 1.76 for online casino/bingo gambling [0.94–3.31], *p* = 0.08, *OR* 2.33 for sports betting [0.59–9.17], *p* = 0.23, *OR* 1.07 for horse race betting [0.58–1.97], *p* = 0.83, and *OR* 0.73 for online poker [0.34–1.55], *p* = 0.41). Also, in logistic regression, self-reported gambling on other operators after the intervention was not significantly associated with gender (*OR* 1.01 for male gender [0.25–4.12], *p* = 0.99), age group (*OR* 0.81 [0.58–1.15], *p* = 0.24), or with any of the specific gambling types assessed (*OR* 1.60 for online casino/bingo gambling [0.86–2.97], *p* = 0.14, *OR* 0.68 for sports betting [0.20–2.35], *p* = 0.54, *OR* 1.77 for horse race betting [0.95–3.29], *p* = 0.07, and *OR* 1.10 for online poker [0.53–2.26], *p* = 0.80).

## Discussion

The present study is one of very few studies assessing gamblers’ own subjective experience of a personalized harm-reducing intervention delivered by a gambling operator. It is one part of a pre-defined follow-up evaluation of a responsible gambling intervention carried out by the state-owned gambling operator of Sweden (Håkansson et al., 2020). Despite limitations related to the limited sample size and a low response rate, this study lends support to a personalized, motivational telephone intervention addressing clients with high-risk gambling practices. In a sample with extensive past-year gambling habits, a large majority reported a favorable impression of the intervention, and a substantial percentage reported, subjectively, that the intervention decreased their gambling practices.

Although the present survey was carried out less than 2 weeks after the telephone intervention, 7% of clients had self-excluded from gambling. It is difficult to know whether this is higher or lower than what could be expected, and it is difficult to draw conclusion from this finding, also due to the low absolute numbers. However, it is of importance to study further whether younger individuals are more likely to self-exclude after this type of motivational intervention, as in the present study. This being said, however, the main finding of the study is likely the fact that four out of five respondents perceived the intervention as mainly favorable, and that only 8% perceived it as negative, and only 7% reported that it increased their gambling. Also, it is of importance to note that individuals who had a favorable impression of the intervention were both more likely to decrease their gambling and less likely to gamble on other operators after the intervention, which further strengthens the impression of the intervention being feasible in the present population.

Also, only a limited minority of respondents (7% of the whole study sample) reported that they gambled on new operators after the telephone intervention. Thus, this data speaks against the fear that this type of intervention – a personalized, harm reduction-oriented intervention to clients with possible gambling problems – would annoy people and to have a counter-productive effect of increased gambling practices or to push gambler towards other operators with less defined responsible gambling practices. Although this is a promising finding, it has to be replicated in larger studies, preferably with higher response rate, in order to rule out the risk that people with a more favorable approach towards the intervention may be more likely to respond to the survey. Also, and despite the fact that the absolute numbers of respondents were low, clients who reported a negative reaction to the call were significantly more likely to report gambling on an operator on which they had never gambled before. This may corroborate with the findings of Ivanova et al. (2019), where a subgroup with gambling problems had more unfavorable attitudes towards responsible gambling practices, and may be a sign of the challenge of intervening in the minority of respondents who gave a more negative description of the intervention. On the contrary, however, clients who reporting being angry or annoyed by the call still were not more likely to report increased gambling. Thus, while the presence of other gambling operators on the market is a reality (Håkansson et al., 2022), with a certain risk of a subgroup changing to other operators after a responsible gambling intervention, they may not necessarily worsen their gambling behavior and they may still retain strategies and patterns of thinking from the motivational conversation. Further longitudinal studies are needed in order to fully outline changes in gambling behaviors in different subgroups reached by thee present type of intervention.

The group of clients assessed in the present study had a high gambling involvement and likely high rates of gambling problems and gambling-related harm. Around one out of six participants reported gambling-related debts and debts involving the national enforcement agency. Data for comparison are few, but a recent online survey of online gamblers in Sweden demonstrated that 9% reported gambling-related borrowing, and that this was highly correlated with gambling problems. In the same study, 8% of online gamblers were involved with the enforcement agency (Håkansson and Widinghoff, 2020). Thus, gambling with borrowed money was more common in the present study than in a comparison group, and although the majority had not such debts, it raises concerns about a sub-group with highly intense gambling practices and indebtedness.

Altogether, the present study adds to and resembles the conclusions by Ivanova et al. (2019), who reported that negative reactions to a responsible gambling intervention were possible but rare. Also, the study adds to the promising findings seen in studies conducted with the Norwegian state-owned gambling operator, and where a telephone intervention was more effective in reducing gambling problems than a control condition or a postal intervention (Jonsson et al., 2019, 2020, 2021). Thus, the study adds to the rationale behind introducing active harm-reducing intervention programs in other gambling operators. In particular, given the low number of respondents who stated a clearly negative experience from the intervention, it appears to be rational for gambling operators to pursue identification of gamblers with hazardous gambling practices, and to carry out a personalized intervention. This intervention can, based on the present findings, initiate a motivational process possibly leading to reduced gambling, self-exclusion, or treatment seeking. Likewise, the study may lend support to policy makers implying regulations on gambling operators on measures to be taken whenever a problematic gambling behavior is detected. For example, the Swedish government has stated, in a gambling act effective since 2019, that gambling operators are to take responsible gambling measures when gambling problems are suspected (Government of Sweden, 2018). While such initiatives need to be implemented and adapted with respect to feasibility and effectiveness, the present study may provide some preliminary evidence that active, personalized interventions as part of that kind of responsible gambling policy are at least unlikely to have a negative impact on gamblers, and more likely may provide an important step in the motivational process of at-risk gamblers.

The present study has limitations. First, based on the defined criteria of high-risk gambling of the present project, a very large percentage of gamblers were men, and for that reason, results or predictors of outcome could not be analyzed for women and men separately. Also, a minority of clients addressed with the intervention could be included in the present study, due to a low response rate. For this reason, results cannot be generalizable to any gambler reached by this intervention, and also cannot be generalizable to individuals with high-risk gambling practices in general. Thus, it cannot be excluded, for example, that subjects with more favorable attitudes towards the intervention were more likely to take the survey. Overall, a low response rate is likely to be a challenge when conducting a self-report survey in close association with a motivation intervention addressing a hazardous gambling behavior, and consequently, the response rate also in the present study was limited. In order to describe the characteristics of included subjects in relation to the attrition rate, the present study sample was compared to what is known about the overall sample of motivational call recipients over a longer period of time, deriving from an eight-month period in 2019 and 2020 from which the authors have access to motivational telephone call data (Håkansson et al., 2020). The subjects in the present study have the same gender distribution, but appear be somewhat older. As in the reference sample, a very large majority are sports bettors. However, it cannot be excluded, based on the nature of the study, that individuals who consented to the present post-intervention survey were more favorable towards the intervention than others (although the opposite, i.e., a willingness to express criticism towards the intervention, also could have been possible).

Also, from a statistical standpoint, the final sample size was likely insufficient for potential sub-group differences to be seen. In regression analyses of a self-reported decrease in gambling, and analyses of gambling on other gambling operators after the intervention, did not display significant predictors for these outcome measures, and it cannot be excluded that such potentially significant interventions would have been hidden by low statistical power. For example, online casino/bingo gambling displayed some non-significant tendency to increase the likelihood of a gambling-decreasing effect after the intervention, and such a potential association would have required a larger study sample to be fully ruled out or confirmed. Likewise, in the non-adjusted analyses of the small groups with rare study outcomes, such associations could not be controlled for other variables in a larger regression analysis. Clients who self-excluded from gambling, and clients who reported initiating gambling on other operators, were significantly younger. These associations, apparently in opposite directions, may possibly indicate a more volatile gambling pattern in the youngest age group. For example, it may be hypothesized that migration between gambling types or between operators may be larger and therefore could explain this pattern in the young, but the testing of such a hypothesis goes beyond the scope of the present study. In brief, larger changes from the present type of intervention can be suspected to occur in the young. Future studies should address larger study samples, in order to better highlight whether these age differences remain after controlling for other variables of relevance.

## Data availability statement

The datasets presented in this article are not readily available because data can be made available upon request to AH, and after review by the data owner, AB Svenska Spel. Requests to access the datasets should be directed to anders_c.hakansson@med.lu.se.

## Ethics statement

The studies involving human participants were reviewed and approved by Swedish Ethics Authority, Sweden. The patients/participants provided their written informed consent to participate in this study.

## Author contributions

AH, KF, MD, and AL contributed to the overall research idea, planned the study together, and carried out data collection. AH carried out the statistical analyses and wrote the draft of the paper. All authors contributed to the manuscript and approved the final version for submission.

## Funding

The present work was financed by the research funding of AH, which comes from AB Svenska Spel and from the regional health care system, and by resources from AB Svenska Spel directly. The funders were not involved in the study design, collection, analysis, interpretation of data, the writing of this article or the decision to submit it for publication.

## Conflict of interest

AH is employed by Lund University, as a full professor in addiction medicine with specialization in problem gambling. His position is financially supported by AB Svenska Spel, which is the government-owned gambling operator of Sweden. All other authors of the paper are employed by AB Svenska Spel.

## Publisher’s note

All claims expressed in this article are solely those of the authors and do not necessarily represent those of their affiliated organizations, or those of the publisher, the editors and the reviewers. Any product that may be evaluated in this article, or claim that may be made by its manufacturer, is not guaranteed or endorsed by the publisher.
